# Craniosynostosis Associated With Novel TUBG1 Mutation (NM_001070.4:c.821C>T) (p.Thr274Ile)

**DOI:** 10.7759/cureus.61132

**Published:** 2024-05-26

**Authors:** Angela S Ash, Kevin M Klifto, Thomas D Willson

**Affiliations:** 1 Division of Plastic and Reconstructive Surgery, Department of Surgery, University of Missouri School of Medicine, Columbia, USA

**Keywords:** epilepsy, pediatric, gene mutation, microcephaly, tubulin, craniosynostosis, neurodevelopment

## Abstract

TUBG1, a tubulin gene, plays an important role in neurodevelopment. Here we describe a case of a novel TUGB1 mutation (NM_001070.4:c.821C>T) (p.Thr274Ile). This patient presented similarly to previous cases with features including microcephaly, epilepsy, and speech and motor delay. Unique characteristics were also present such as trigonocephaly, tethered frenulum, scoliosis, nystagmus, and a concurrent FBXW7 mutation. This case expands our breadth of knowledge on TUBG1 genotypic and phenotypic variation. However, further work is needed to fully understand this rare mutation and the associations between TUBG1 and FBXW7 mutations.

## Introduction

Microtubules provide the structure and forces needed by neurons to develop axonal and dendritic processes [1]. Tubulin comprises a large portion of the microtubules [1]. Thus, tubulin genes affect several aspects of cortical development [2]. Mutations in tubulins and microtubule-associated proteins have been linked to a large spectrum of cortical malformations [3]. In humans, γ-tubulin is largely conserved and is encoded by two genes, namely γ-tubulin 1 and γ-tubulin 2 (TUBG1 and TUBG2) [3]. TUBG1 is significantly upregulated during fetal brain development, participating in centrosome formation and the crucial role of microtubule nucleation [4]. If this process is disrupted, microtubule-dependent mitosis and brain development are affected [5]. Various TUBG1 mutations have exhibited pleiotropic phenotypic presentations [2,5-6]. Clinical features can include motor dysfunction, intellectual disability, epilepsy, and cortical dysgenesis [2,5-6]. As of 2023, there have been 13 published cases of TUBG1 with nine different mutations reported, with one additional case that was retracted due to unknown causes [2,5-6]. We describe the 14th variant along with a novel 10th mutation.

## Case presentation

The patient was born prematurely at 34.1 weeks gestational age via spontaneous vertex vaginal delivery, phenotypically and genotypically female as a 46 XX karyotype. Limited prenatal care was reported, with a pregnancy confirmed 40 days prior to delivery. In-utero ultrasound, performed 14 days prior to delivery, first identified microcephaly, skull asymmetry, and a thickened nuchal fold. Findings prompted amniocentesis, which resulted in subsequent preterm premature rupture of membranes. Meconium-stained amniotic fluid was noted upon rupture of membranes. The pregnancy was complicated by a right humeral fracture during delivery. The patient required resuscitation and positive pressure ventilation following delivery.

On day one following birth, cranial ultrasound demonstrated bilateral caudothalamic and choroid plexuses cystic structures with a globular appearance of the choroid plexus and ventriculomegaly. An anteroposterior (AP) radiograph of the skull demonstrated that cranial sutures were widely open with overlying parietal scalp swelling but no evidence of craniosynostosis. Additionally, a chest radiograph demonstrated bilateral hazy opacities consistent with respiratory distress syndrome.

**Figure 1:**
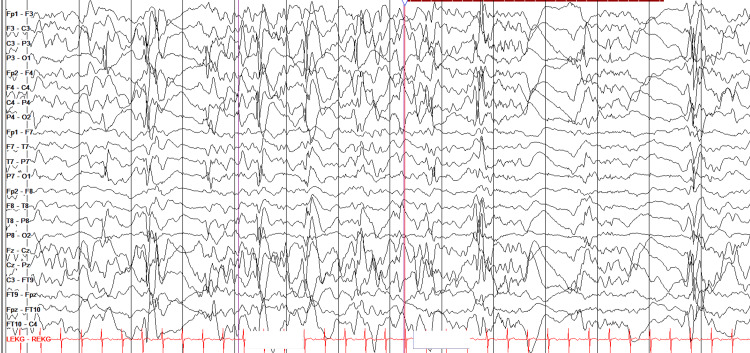
Electroencephalogram (EEG) results This EEG demonstrates significant suppression and slowing of ongoing background activity with notable medium to high amplitude discharges with intermixed polyspike-wave discharges. This pattern is consistent with Ohtahara syndrome.

On day two, the patient experienced seizures associated with Ohtahara syndrome, diagnosed via video electroencephalogram (EEG) (Figure 1). The decreased EEG activity led to the suspicion of polymicrogyria. Cranial features included hypotelorism, upslanting palpable fissures, micrognathia, and a broad nose. Whole-exome sequencing confirmed the patient had a de novo mutation of TUBG1 (NM_001070.4:c.821C>T) (p.Thr274Ile), inherited in a heterozygous manner, likely as a pathogenic variant. Additionally, the patient was found to have a mutation of FBXW7 (NM_033632.3:c.1045A>G) (p.Ser349Gly), inherited in a heterozygous manner from the mother, with uncertain significance. Of note, family history was significant for asthma, attention deficit hyperactivity disorder (ADHD), and depression. The family history was negative for clinical features that were similar to this patient's presentation. On day three, cranial ultrasound comparisons were unchanged from day one. 

On day eight, magnetic resonance imaging (MRI) of the brain demonstrated low periventricular white matter volumes and abnormally smooth gyri and sulci, consistent with microlissencephaly. Thinning of the corpus callosum was present, along with brainstem and cerebellar vermian hypoplasia, ventriculomegaly, and hypotelorism.

At 12 months, re-examination performed by neurology showed upslanting palpable fissures, a broad nose, a narrow forehead, facial asymmetry, and micrognathia. Nystagmus with central hypotonia and appendicular spasticity was present. The patient had a global developmental delay but was able to say a few words. Seizures occurred daily. Her EEG was clinically consistent with Lennox-Gastraut syndrome at this time.

**Figure 2:**
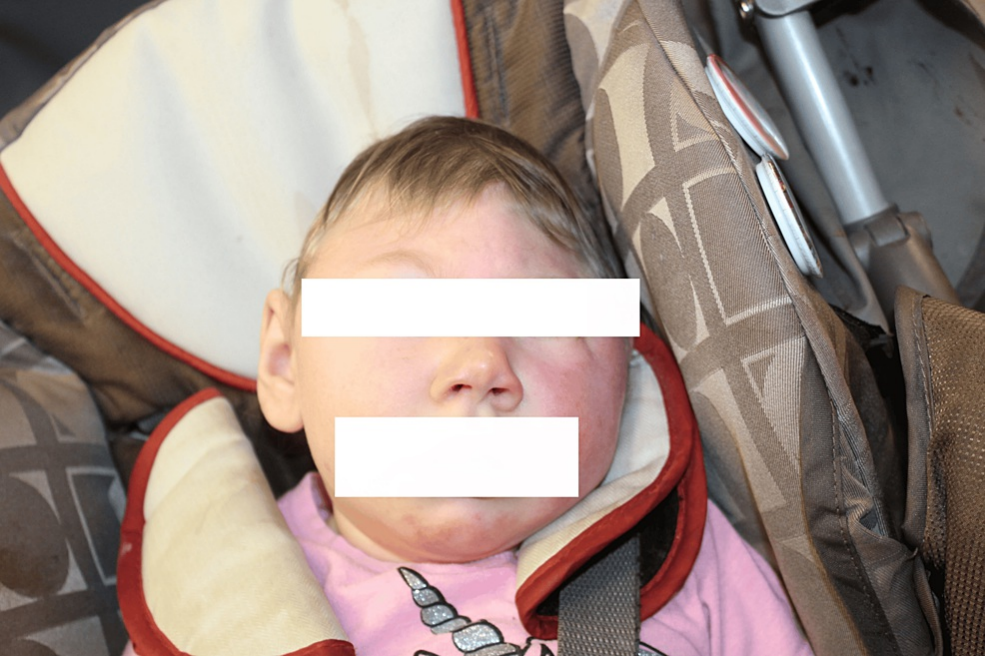
Cranial image (frontal view) Craniofacial deformities pictured include trigonocephaly with an osseous ridge, bitemporal narrowing, a narrow forehead, and broad nose.

**Figure 3:**
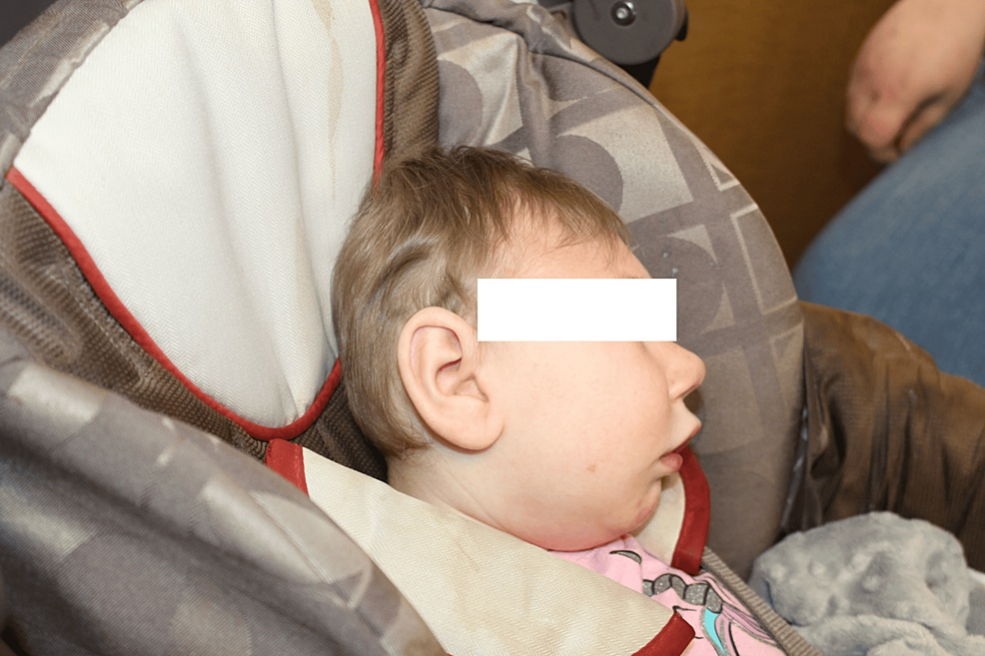
Cranial image (profile view) Craniofacial deformities pictured include craniosynostosis, low-set ears that are prominent relative to the microcephaly, and micrognathia.

**Figure 4:**
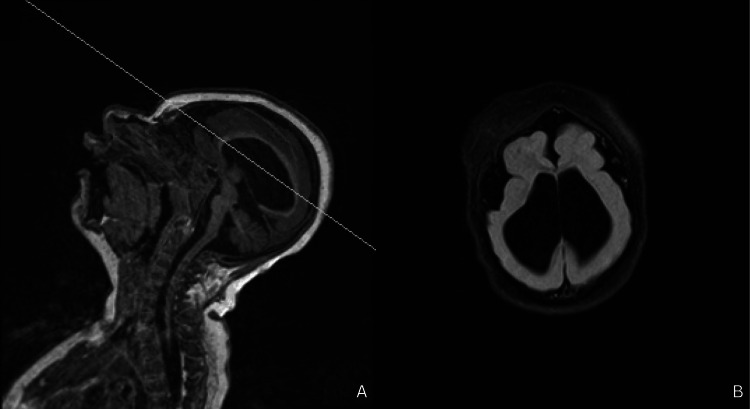
MRI brain (sagittal and axial view) A: On sagittal view, ventriculomegaly, brainstem hypoplasia, and cerebellar vermis hypoplasia can be appreciated; B: On axial view, there is a notably thin corpus callosum, lateral ventriculomegaly, and diffuse agyria that is more prominent posteriorly.

At 15 months, the patient was diagnosed with oropharyngeal dysphagia after aspiration was confirmed following a barium swallow study. A gastrostomy tube was placed, and a Ladd’s procedure was performed for feeding dependence and midgut malrotation. At 16 months, she presented to the plastic and reconstructive surgery clinic following concerns of microcephalic trigonocephaly (Figures 2, 3). The patient remained in the 0th percentile for head circumference with a measurement of 33 cm. The physical exam was notable for trigonocephaly, indicated by the premature closure of the metopic suture, resulting in the inability of the frontal bones to grow laterally, thus forming a triangular forehead with an osseous ridge. Her anterior fontanelle was closed. The face and occipital condyles were symmetric bilaterally, with bitemporal narrowing, forehead narrowing, and the absence of squamosal bulging. There were no restrictions on the neck's range of motion. The ears were symmetric and well-formed, with no abnormalities. Intra-oral examination revealed a normal palate with no clefting; however, a tethered frenulum was noted. Evaluation by orthopedic surgery at this time also noted neuromuscular scoliosis. The repeat MRI of the brain performed at 19 months was overall unchanged from the MRI on day eight (Figures 4A, 4B). 

The patient continues to be monitored. The mother wanted to avoid surgeries unless they were medically necessary. Beyond 12 months, patients typically do not have neurological benefits following metopic craniosynostosis surgery [7]. The patient was referred to pediatric ophthalmology to assess for increased intraocular pressure and intracranial pressure. These features, if present, may warrant corrective surgery [8].

## Discussion

This is the first reported case of the TUBG1 gene mutation (NM_001070.4:c.821C>T) (p.Thr274Ile), a single nucleotide missense mutation [9]. As of 2023, there have been 13 published cases of TUBG1 mutations, with nine different mutations reported (Table 1) [2,5-6]. This patient presents with a novel mutation. TUBG1 and its paralog, TUBG2, are both highly expressed in the hippocampus, thalamus, cerebellum, and cerebral cortex; however, TUBG1 exhibits greater expression compared to TUBG2 [5]. These proteins are part of a larger tubulin superfamily, but unlike α-tubulin and β-tubulin, γ-tubulin is absent from the microtubule lattice; rather, it is necessary for the polymer formation of proteins, α-tubulin and β-tubulin [5]. To do so, γ-tubulin localizes to the centrosome during interphase [5]. While proteins within the tubulin superfamily are highly similar, the phenotypic distinctions associated with tubulin isotype variants support the hypothesis that each tubulin has a unique function [10].

**Table 1:** Genotype, phenotype, and EEG findings of reported TUBG1 variants N/A: not applicable; SD: standard deviation; CP: cerebral palsy; ASD: atrial septal defect; y: years; mo: months; d: days *Study retracted due to unknown causes

	Patient 1	Patient 2	Patient 3	Patient 4	Patient 5	Patient 6	Patient 7	Patient 8	Patient 9	Patient 10	Patient 11	Patient 12	Patient 13	Patient 14	Patient 15
Study	Poirier 2013	Poirier 2013	Poirier 2013	Brock 2018	Brock 2018	Brock 2018	Brock 2018	Brock 2018	Brock 2018	Brock 2018	Brock 2018	Yuen 2019	Yuen 2019	Shen 2021*	This study
Nucleotide sequence variation	c.1160T>C	c.275A>G	c.991A>C	c.63C>A	c.985G>T	c.776C>T	c.776C>T	c.776C>T	c.776C>T	c.769A>T	c.776C>T	c.202G>A	c1021C>T	c.751A>T	c.821C>T
Protein sequence variation	p.Leu387Pro	p.Tyr92Cys	p.Thr331Pro	p.Phe21Leu	p.Asp329Tyr	p.Ser259Leu	p.Ser259Leu	p.Ser259Leu	p.Ser259Leu	p.Ile257Phe	p.Ser259Leu	p.Asp68Asn	p.Arg341Trp	p.Asn251Tyr	p.Thr274Ile
Zygosity	--	--	--	Heterozygous	Heterozygous	Heterozygous	Heterozygous	Heterozygous	Heterozygous	Heterozygous	Heterozygous	--	--	Heterozygous	Heterozygous
Mode of inheritance	De novo	De novo	Paternal DNA unavailable	De novo	Paternal DNA unavailable	De novo	De novo	Parental germline mosaicism	De novo	De novo	De novo	De novo	De novo	De novo	Undetermined
Mutation effect	Leucine to proline within a highly conserved residue within an α-helix found in the C-terminal domain	Tyrosine to cysteine within a highly conserved residue in the vicinity of the GTPase domain	Threonine to proline within a highly conserved residue in an α-helix within the y-y protein interaction domain found in the C-terminal domain	Phenylalanine to leucine within the GTPase domain	Aspartate to tyrosine within a highly conserved residue in the C-terminal domain; found on the TUBG1 surface	Serine to leucine within a highly conserved residue found in the C-terminal domain	Serine to leucine within a highly conserved residue found in C-terminal domain	Serine to leucine within a highly conserved residue found in the C-terminal domain	Serine to leucine within a highly conserved residue found in the C-terminal domain	Isoleucine to phenylalanine within a highly conserved residue found in the C-terminal domain	Serine to leucine within a highly conserved residue found in the C-terminal domain	Aspartate to asparagine within a highly conserved residue found in the GTP-binding pocket	Arginine to tryptophan within a highly conserved residue found in the C-terminal domain, required for dimerization of γ-tubulin	--	--
Additional mutations	None	None	None	None	None	None	None	None	None	None	None	None	None	None	FBXW7
Findings associated with additional mutations	N/A	N/A	N/A	N/A	N/A	N/A	N/A	N/A	N/A	N/A	N/A	N/A	N/A	N/A	Intellectual disability, language disorder, hypotonia, macrocephaly, microcephaly, cryptorchidism, feeding difficulties, constipation, large cerebellar vermis, tonsillar ectopia, thick callosal genu, thin corpus callosum, subcortical white matter hyperintensities, delayed myelination, cardiac anomalies, palatal/uvular/laryngeal anomalies
Sex	F	M	F	M	M	F	F	F	M	M	F	F	M	F	F
Age at presentation	21 y	18 mo	31 y	33 y	21 y	19 mo	14 y	11 y 6 mo	9y 6mo	15 y	18 mo	10 y	6 mo	8 y 9 mo	16 mo
Epilepsy	Yes	Yes	Yes	Yes	Yes	Yes	Yes	Yes	Yes	Yes	Yes	No	Yes	Yes	Yes
Seizure age of onset	--	--	--	36 mo	--	6 mo	6 mo	4 mo	--	3 y 11 mo	5 mo	N/A	< 1 hr of life	1 y	2 d
Types of seizures	Early-onset	Infantile spasms	Early-onset	Tonic-atonic-myoclonic	Partial complex; versive seizure, myoclonic	--	Tonic-clonic	Generalized tonic-clonic	--	--	Focal, versive	N/A	Focal left-sided clonic activity with secondary bilateral synchrony	--	Infantile spasms
Refractory epilepsy	Yes	Yes	Yes	--	Yes	--	--	--	--	--	--	N/A	No	Yes	No
EEG findings	--	--	--	--	--	--	--	--	--	--	--	Intermittent interictal sharp waves over the right central temporal areas during drowsiness and sleep, indicating a tendency toward focal onset seizures; sporadic non-specific generalized paroxysmal delta activity during sleep	Diffuse suppression and excessive left central sharp waves	Increased background delta activity; sharp waves and sharp slow waves over the frontal and occipital cortex bilaterally	Hypsarrhythmia; high amplitude, disorganized and poorly modulated mixed frequency of rhythmic delta with superimposed high amplitude generalized spikes which persist throughout the recording. Periodic pseudo-burst suppression patterns seen. Frequent myoclonic body movement seen, and some associated with generalized spikes
Cranial features	Microcephaly	Microcephaly	Normocephalic	Normocephalic	Microcephaly	Microcephaly	--	Microcephaly	--	Microcephaly	--	Microcephaly	Microcephaly	--	Microcephaly, trigonocephaly
Head circumference cm (SD)	(< -5.5)	(< -4)	(< -1)	57 cm	53.1 cm (< -2.6)	(< -3.5)	--	47.5 cm at 6y 6mo (< -3.3)	--	51.3 cm at 13 y (< -2.5)	--	46 cm (< -2.1)	(< -1.9)	--	33 cm
Intellectual disability	Severe	Severe	Moderate	Severe	Severe	--	--	Moderate	Moderate	Moderate (FS IQ-score 44)	Severe	Moderate	Moderate global delay	Severe	Global delay
Speech/language development	--	--	--	No speech, only sounds	Nonverbal	Delayed	Nonverbal	Speaks 50 words	Nonverbal	Speaks 5-6 word sentences	Nonverbal	Normal	Moderate global delay	Can repeat words, cannot count or communicate	Can make sounds and say a few words
Motor dysfunction	Spastic quadriplegia	Spastic quadriplegia	Moderate CP	Spastic quadriplegia; Assisted ambulation	Spastic quadriplegia	Delayed	Gait instability	--	--	--	Delayed	No	Axial hypotonia and appendicular hypertonia	Gait instability	Axial hypotonia and appendicular hypertonia
Feeding	--	--	--	Assisted feeding	Gastrostomy	--	--	Drooling	--	--	--	--	--	--	Gastrostomy
Other abnormalities	--	--	Cataracts	--	--	--	--	Strabismus	--	--	--	Prominent ears, mouth tenting, bilateral 5th digit clinodactyly	Upslanting palpebral fissures, central hypothyroidism, small optic nerves, secundum ASD	Short stature, left auricle deformity, pectus excavatum	Scoliosis, upslanting palpable fissures, broad nose, micrognathia, nystagmus, narrow forehead, tethered frenulum

Neurodevelopment

Phenotypic features shared by most of the patients with TUBG1 mutations include moderate to severe intellectual disability, motor impairment, and speech delays ranging from nonverbal to moderately delayed speech [2,5-6]. Due to the young age at diagnosis of this patient, intellectual disability and speech cannot be comprehensively assessed; however, she was noted to have a global developmental delay with the ability to make sounds and say a few words at 12 months old. It should be noted that, depending on severity, intellectual disability may not appear initially. A previous patient with a TUBG1 mutation did not have an apparent intellectual disability until six years of age [6]. Among these 14 patients, motor dysfunction ranged from normal to unstable gait to spastic quadriplegia. This patient presented with axial hypotonia and appendicular spasticity, as did one other patient with a TUBG1 mutation [6].

Phenotypic abnormalities

Our patient presented with oropharyngeal dysphagia requiring a gastrostomy. Three published descriptions of patients had associated feeding issues, such as assisted feeding or drooling, with one patient also requiring a gastrostomy [5]. Microcephaly was reported in five of 14 published patients, including ours. Other abnormalities reported in published patients include short stature, ear deformity, pectus excavatum, prominent ears, tented mouth, clinodactyly, cataracts, strabismus, upslanting palpebral fissures, secundum atrial septal defect, and central hypothyroidism [2,5-6]. Our patient presented with unique additional phenotypic characteristics not previously reported among other patients with TUBG1 mutations, such as trigonocephaly, nystagmus, scoliosis, and a tethered frenulum.

MRI findings

On MRI, most patients had a ranging severity of reduced white matter with ventriculomegaly. Gyral patterns were decreased in all patients, ranging from pachygyria to lissencephaly, with posterior gyri affected more severely compared to anterior gyri in most patients. Other reported MRI abnormalities include thickened cerebellar cortex, dysmorphic corpus callosum, hippocampal malrotation, brainstem hypoplasia, and cerebellar malformation (Table 2) [2,5-6].

**Table 2:** MRI findings of reported TUBG1 variants y: years; mo: months; d: days; P>A: posterior>anterior *Study retracted due to unknown causes

	Patient 1	Patient 2	Patient 3	Patient 4	Patient 5	Patient 6	Patient 7	Patient 8	Patient 9	Patient 10	Patient 11	Patient 12	Patient 13	Patient 14	Patient 15
Study	Poirier 2013	Poirier 2013	Poirier 2013	Brock 2018	Brock 2018	Brock 2018	Brock 2018	Brock 2018	Brock 2018	Brock 2018	Brock 2018	Yuen 2019	Yuen 2019	Shen 2021*	This study
Age at MRI	--	--	--	36 y	11 y	1 y 6 mo	12 mo	13 y 7 mo	2 mo	6 y	9 y	9 y	13 d	7 y	8 d
Gyral pattern	Pachygyria / Agyria	Pachygyria / Agyria	Pachygyria	Pachygyria	Agyria	Pachygyria	Pachygyria	Pachygyria	Pachygyria	Pachygyria	Pachygyria	Pachygyria	Agyria	Pachygyria	Agyria
Cerebellar cortex thickness	Thick	Thick	--	--	--	10 - 13 mm	13 - 15 mm	> 15 mm	> 15 mm	6 - 10 mm	--	--	--	Thick	9 - 10 mm
Gradient	P>A	P>A	P>A	P>A	P>A	P>A	P>A	P>A	P>A	P>A	P>A	P>A	P>A	P>A	P>A
White matter	Mildly reduced	Severely reduced	Normal	Enlarged perivascular spaces	Severely reduced	Mildly reduced	Mildly reduced	Normal	Normal	Mildly reduced	Mildly reduced	--	Reduced	--	Severely low volume of periventricular white matter predominantly within frontal lobes
Lateral ventricles	Mildly enlarged	Mildly enlarged	Normal	Enlarged posterior horns	Severely enlarged	Mildly enlarged	Mildly enlarged	Enlarged posterior horns	Mildly enlarged	Mildly enlarged posterior horns	Mildly enlarged	--	Enlarged	Enlarged	Diffuse moderate ventriculomegaly with prominence of extra-axial subarachnoid spaces
Corpus callosum	Thin	Dysmorphic, thick	Dysmorphic, thick	Normal	Thin	Normal	Normal	Normal	Thin	Thin	Thin	Normal-thick	Normal	Dysmorphic	Thin
Basal ganglia	Normal	Normal	Normal	Normal	Dysplastic	Normal	Normal	Normal	Normal	Dysplastic	Dysplastic	--	Hypoplasia of lentiform nuclei and thalami; decreased myelination of posterior limbs of internal capsule	--	Dysplastic
Hippocampus	--	--	--	Malrotation	--	Normal	Normal	Normal	Normal	Normal	Normal	--	--	--	Malrotation
Brainstem	Normal	Normal	Normal	Normal	Hypoplasia	Normal	Normal	Normal	Normal	Normal	Normal	--	--	--	Hypoplasia
Cerebellar cortex	Normal	Normal	Normal	Normal	Normal	Normal	Normal	Normal	Normal	Normal	Normal	--	Small cerebellum	Poor structure	Small cerebellum
Cerebellar white matter	Normal	Normal	Normal	Normal	Normal	Normal	Normal	Normal	Normal	Normal	Normal	--	Small cerebellum	Poor structure	Small cerebellum
Vermis	Normal	Normal	Normal	Normal	Hypoplasia	Normal	Normal	Normal	Normal	Normal	Normal	--	--	--	Hypoplasia

Epilepsy

Epilepsy is another clinical feature commonly shared by patients with TUBG1 mutations. Only one of the 14 published patients was not reported to have epilepsy [6]. Our patient had EEG findings consistent with Ohtahara syndrome at birth. Ohtahara syndrome is characterized by tonic spasms and partial seizures that onset within the first few months of life and alternating high-voltage bursts and flat suppressions on the EEG [11]. Remarkably, this syndrome evolves over time; approximately 75% of children with Ohtahara syndrome transform into West syndrome (WS) during three to six months of age and further from WS to Lennox-Gastaut syndrome at one to three years of age, as was seen in this patient [11].

FBXW7

**Figure 5:**
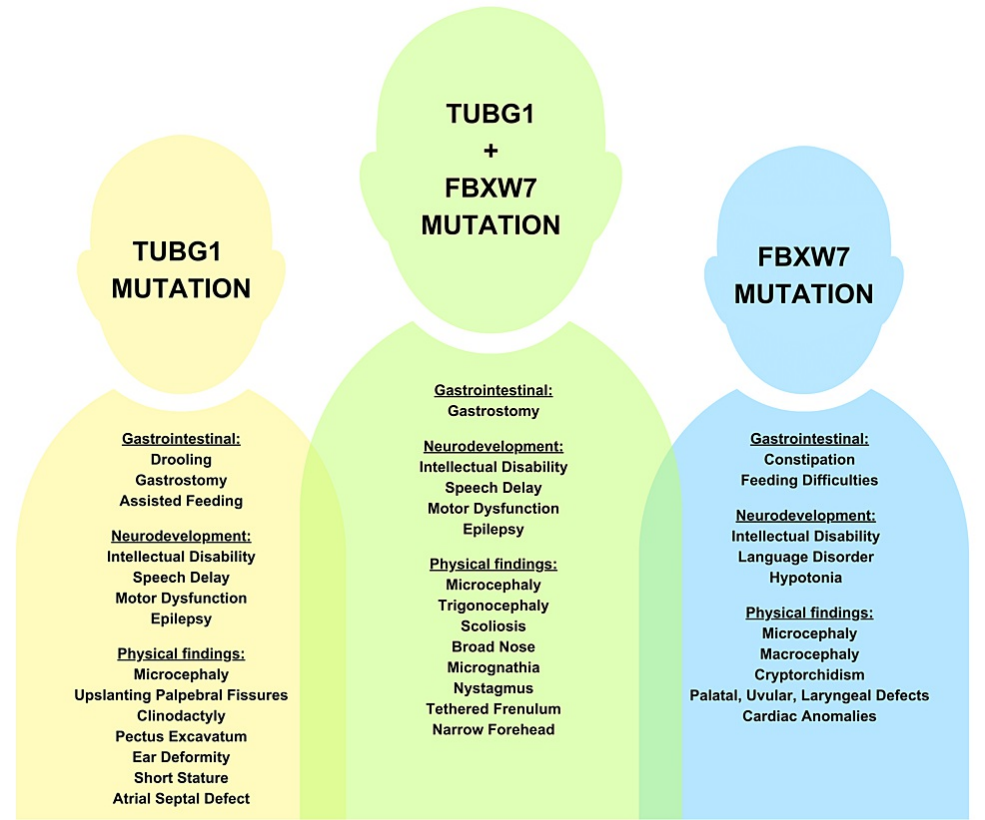
Comparison of TUBG1, FBXW7, and TUBG1 + FBXW7 presentations There is a large overlap between phenotypic presentations of patients with TUBG1 and FBXW7 mutations. Involvement of gastrointestinal and neurological systems was seen in these patients, along with similar physical deformities. * Image is created by the authors

Additionally, this patient presents with a germline heterozygous FBXW7 mutation (NM_033632.3:c.1045A>G) (p.Ser349Gly). This is the first reported case of a TUBG1 mutation co-occurring with a FBXW7 mutation. FBXW7 is a tumor suppressor gene that is structurally composed of seven WD40 repeat domains, a dimerization domain, and an F-box domain [12]. This gene is involved in the recruitment of Skp1-cullin 1-F-box (SCF), an E3 ubiquitin ligase complex [12,13]. The clinical phenotype of heterozygous FBXW7 pathogenic variants is characterized by neurodevelopmental disability, including global developmental delay, intellectual disability ranging from borderline to severe, language disorder, and hypotonia [14]. A review of 35 individuals with germline FBXW7 variants reports seizures (22%), feeding difficulties and constipation (45.7%), macrocephaly (28.6%), microcephaly (5.7%), palatal, uvular, or laryngeal anomalies (31.4%), cardiac anomalies (31.4%), and cryptorchidism (19.2%) [14]. MRIs of this same cohort exhibit counts of large cerebellar vermis’ with tonsillar ectopia, thick callosal genu, thinned corpus callosum, subcortical white-matter hyperintensities, and severely delayed myelination [14]. Heterozygous truncating FBXW7 variants have been reported in four individuals with Wilms tumors and a de novo non-synonymous FBXW7 mutation in a child with a rhabdoid tumor [15]. However, no individuals with neurodevelopmental features in published cohorts have a history of cancer [14]. As it relates to this case, the pathogenicity of their variant is unknown. While there is an overlap in phenotypic presentation between FBXW7 mutations and TUBG1 mutations, it is unclear if this FBXW7 mutation has contributed to the patient’s phenotypic presentation (Figure 5). Further research is needed to clarify the possible link between germline FBXW7 variants and TUBG1 variants.

## Conclusions

We report the first published description of a patient with the novel TUBG1 mutation (NM_001070.4:c.821C>T) (p.Thr274Ile) and co-occurring FBXW7 mutation (NM_033632.3:c.1045A>G) (p.Ser349Gly). TUBG1 mutations have been linked to a spectrum of cortical malformations, including microcephaly, polymicrogyria, pachygyria, and agyria. These disorders are often associated with intellectual disabilities, seizures, and motor impairments. While this case expands our breadth of knowledge on TUBG1 genotypic and phenotypic variation, further work is necessary to completely understand this rare genetic mutation and its potential associations with FBXW7 mutations.
